# Risk Factors for Oral Health in Anorexia Nervosa: Comparison of a Self-Report Questionnaire and a Face-to-Face Interview

**DOI:** 10.3390/ijerph18084212

**Published:** 2021-04-16

**Authors:** Hélène Rangé, Alice Pallier, Aminata Ali, Caroline Huas, Pierre Colon, Nathalie Godart

**Affiliations:** 1 Department of Periodontology, Université de Paris, UFR of Odontology, Service of Odontology, Rothschild Hospital, AP-HP, 75012 Paris, France; alicepallier@hotmail.fr; 2 Université de Paris, UR 2496 Orofacial Pathologies, Imaging and Biotherapies, 92120 Montrouge, France; 3 Paris-Saclay University, Paris Sud University, UFR Simone Veil, UVSQ, CESP, INSERM, 94800 Villejuif, France; aminata_ali@yahoo.fr (A.A.); caroline.huas@fsef.net (C.H.); nathalie.godart@fsef.net (N.G.); 4 Fondation Santé des Etudiants de France (FSEF), 75014 Paris, France; 5 Department of Restorative Dentistry and Endodontics, Université de Paris, UFR of Odontology, Service of Odontology, Rothschild Hospital, AP-HP, 75012 Paris, France; pierre.colon@aphp.fr; 6 Laboratoire Multimatériaux et Interfaces, Université Claude Bernard Lyon 1, UMR CNRS 5615, 69622 Villeurbanne, France; 7 Institut Mutualiste Montsouris, 75014 Paris, France

**Keywords:** anorexia nervosa, risk factors, oral health, oral hygiene, self-report, questionnaire

## Abstract

Behavioral, nutritional, and local risk factors for oral health are frequent in people with anorexia nervosa. However no self-report questionnaire is available for screening in clinical practice or for research purposes. The objective of this study was to design a questionnaire to identify risk factors and symptoms of oral diseases and to test its reliability as a self-report form among people with anorexia nervosa. A 26-item questionnaire was designed based on a sound literature review performed by a group of dentists, psychiatrists, and epidemiologists specialized in the field of eating disorders. Sixty-nine anorexia nervosa inpatients (mean age 18.72 ± 5.1) were included from four specialized units. The questionnaire was first self-reported by the patients, then the same questionnaire was administrated by a dentist during a structured face-to-face interview as the gold standard. The concordance between the two forms was evaluated globally and item per item using Cohen’s kappa statistical tests. The overall concordance between the self-report questionnaire and the face-to-face structured interview was 55%. Of the 26 items, 19 showed significant concordance. Items relating to water intake, extracted teeth, gingival status, and oral hygiene had the best concordance (all kappa coefficients > 0.4). A questionnaire that identifies risk factors and symptoms of oral diseases in anorexia nervosa was developed and tested. The 26-item form of the questionnaire (long version) is moderately reliable as a self-reported form. A short version of the questionnaire, including the 10 most reliable items, is recommended for oral risk assessment in patients with anorexia nervosa. The clinical value of the self-administered questionnaire remains to be evaluated.

## 1. Introduction

Anorexia nervosa (AN), one of the most frequent eating disorders [1] is frequently associated with oral manifestations, such as erosive tooth wear, dental caries, and periodontal diseases [2,3,4,5]. Erosive tooth wear is defined as a superficial loss of hard dental tissue due to a chemical process, without bacterial implication [6]. Erosion develops under the influence of acid (pH value of 5.5), which can be of extrinsic or intrinsic origin. Intrinsic acidity results from a combination of gut disorders such as gastroesophageal reflux, self-induced vomiting, and merycism (rumination syndrome). Extrinsic acidity is linked to the consumption of acidic food such as fruits, and soft drinks. Dental caries result in tooth demineralization by acid production caused by the bacteria-induced fermentation of sugar. Despite a considerable decrease observed in the last few decades, the prevalence of dental caries remains a health issue of concern, both among adults and children, and it is particularly high among patients with AN [3]. Periodontal diseases are chronic multifactorial inflammatory diseases associated with dysbiotic dental plaque, including gingivitis and periodontitis [7,8]. Gingival recessions are defined as an apical shift of the gingival margin caused by different conditions/pathologies, leading to root surface exposure to the oral environment [9]. Dental caries and periodontal diseases share common risk factors related to carbohydrate intake, poor oral hygiene, and hyposalivation [10]. Oral diseases lead to pain, dysfunction, and aesthetic issues having an adverse impact on the self-esteem and quality of life in people with eating disorders [11]. Moreover, they visit their dentist less frequently than the general population [12,13,14]. Survey studies have identified several barriers to prevention and dental care, both in patients with eating disorders (dental fear, denial), and in oral health care providers (lack of training and network) [14,15,16,17,18]. Therefore, self-screening of oral risk factors and symptoms using a questionnaire should be part of the routine healthcare circuit for people with AN [19]. However, as far as we know, there is no easy to handle questionnaire available to evaluate risk factors and symptoms of oral diseases to be used by AN patients themselves. The objectives of the study were: (1) to design such a questionnaire and, (2) to test its reliability as a self-report questionnaire (SRQ-form) in a sample of AN inpatients by cross-referencing the results with those obtained using the same instrument administrated as a hetero-report questionnaire (Q-form) by a specialized dentist.

## 2. Materials and Methods

### 2.1. Context of the Study

A senior multidisciplinary expert group in eating disorders involved in the Fédération Française Anorexie Boulimie, collaborated on a trans-disciplinary clinical study, entitled EVHAN (evaluation of hospitalization for anorexia nervosa; Eudract number: 2007-A01110-53; registered in clinical trials). The study protocol was approved by the Ile-de-France III Ethics Committee and the CNIL (Commission nationale de l’informatique et des libertés).

The inclusion criteria were: (1) age between 8 to 65 years old; (2) hospitalized for AN (DSM-IV-TR criteria) [20], and therefore unable to be treated otherwise because of their somatic state (body mass index < 15 kg/m^2^) or their mental state; (3) written informed consent of their parents for minors and adults living with their parents; (4) written informed consent of the patient obtained; and (5) being affiliated with social security. The exclusion criteria were: (1) patients with a comorbidity that may impact the oral health, like diabetes mellitus and inflammatory bowel disease; (2) not being proficient in the French language.

### 2.2. Questionnaire Design

The questionnaire was drawn up by a group of specialized dentists, psychiatrists, and epidemiologists from the literature related to risk factors for oral diseases in eating disorders patients and related to erosive tooth wear in the general population [12,13,21,22,23,24,25,26]. A comprehensive questionnaire was designed following the structure of a standard dental examination divided into 7 themes: dental history, lifestyle, nutritional status, oral hygiene, salivary flow, gingival status, and dental status (Table 1).

The 26-item SRQ-form was pre-tested in a sample of 20 consecutive individuals with AN attending a dental visit at Rothschild Hospital (Paris, France) to assess the comprehensibility of the items. Pilot patients were then face-to-face interviewed by the dental examiner (PC) about the overall questionnaire comprehensibility and clarity. No major revision of the questionnaire was requested following the cognitive evaluation.

### 2.3. Questionnaire Evaluation

The reliability evaluation of the SRQ-form was tested against the Q-form delivered during a structured face-to face interview, following a previously published methodology [27,28]. The patients filled in the SRQ-form at the end of their eating disorder hospitalization in four Parisian centers (Institut Mutualiste Montsouris, Sainte-Anne Hospital, Maison de Solenn, Robert Debré Hospital, and Paul Brousse Hospital). Then a dental structured face-to-face interview and clinical examination were organized using the same questionnaire filled in by a specialized dentist (PC) at the Rothschild Hospital.

### 2.4. Statistical Analysis

A concordance analysis was conducted between data from the SRQ-form and the Q-form. Cohen’s kappa coefficient was used to assess agreement between the two methods of administration of the questionnaire: self-administered and hetero-administered. Kappa coefficients of >0.6, 0.6–0.41, 0.4–0.21, and ≤0.2 were considered substantial, moderate, fair, and slight, respectively, according to Landis and Koch’s criteria [29]. A *p*-value less than 0.05 was considered statistically significant. The statistical software SPPS 20.0 (IBM, Armonk, NY, USA) was used.

## 3. Results

### 3.1. Description of the Participants

Sixty-nine female consecutive inpatients agreed to participate and were included in the present study. The mean age of the AN inpatients was 18.72 (SD = 5.1), and 64.1% were under 18 years old. Concerning the diagnosis of AN, 50.9% of the inpatients presented a restrictive type and 49.1% a binging–purging type. The mean duration of AN was 3.83 years (SD = 6.13). The mean BMI of the study sample was 14.25 (SD = 1.51). In the interviewed inpatients, dry mouth, gum bleeding after toothbrushing, and tooth hypersensitivity were reported by 33.3%, 47.3%, and 37.5%, respectively.

### 3.2. Reliability Evaluation of the Self-Report Questionnaire Form

All patients agreed to answer the SRQ-form, however 5 questionnaires were not completed (missing data). The remaining 64 inpatients agreed to continue with the study, and completed the Q-form administered by the specialized dentist after their hospitalization. The results of the concordance analysis are described in Table 2.

From the 26 items under scrutiny, 19 were significantly concordant with a *p*-value < 0.05. No questions from the theme related to salivary flow retrieved statistical agreement. The overall agreement of the questionnaire was fair at 55.55%. The agreement significance varied from excellent to poor (100% to 12%), depending on the item (data not shown). The best agreements between the SRQ-form and the Q-form were obtained for the items related to water intake (kappa coefficient = 0.655) and extracted teeth (kappa coefficient = 0.638). Eight items presented moderate agreement (between 0.41–0.6), 4 from the lifestyle theme: sport activity/body appearance, 2 from the oral hygiene theme: amount of toothbrushing/type of toothbrush, and 2 from the gingival status theme: gum bleeding/root exposure. Nutritional status items (except for water intake) and dental status showed fair agreement (Table 3).

## 4. Discussion

Identifying oral health status in people with AN is essential to provide resources to patients for prevention and dental care. Being able to collect this information will become increasingly important for psychiatrists and physicians in a multidisciplinary approach to eating disorder treatment. Therefore, a questionnaire for self-screening of oral risk factors and symptoms in people with severe AN was developed and its reliability was tested. Qualitative data covering seven themes depicted factors influencing oral health status: dental history, lifestyle, nutritional status, oral hygiene, salivary flow, gingival status, and dental hygiene were identified. In the present study, gum bleeding after toothbrushing was the most frequently reported oral health symptom, with 47.3% among AN inpatients. Tooth hypersensitivity was also frequently reported by 37.5% of the patients. A case control study in eating disorders patients, including both AN and bulimia nervosa, published similar results, with 41% reporting dental problems [21]. However, only 33% among AN inpatients in our study self-reported to feel a dry mouth, in comparison to the 52% and 92% previously reported [21,24]. The discrepancy observed between our results and those from Viera Esteves and coworkers may be explained by the study population (AN inpatients only versus a mixt cohort of mainly bulimia nervosa patients with few anorexia nervosa patients). A methodological difference should also be highlighted because they used a specialized five-item questionnaire developed for evaluating subjective feeling of mouth dryness (xerostomia) [30], whereas two questions where used in the present questionnaire.

As far as we know, there has been no study that compared the reliability of a self-report questionnaire on oral health in eating disorders with a face-to-face interview as a gold standard. Prior observational studies constructed various questionnaires, used during face-to-face interviews only [12,13,21,22]. The recently published questionnaire in research from Norway and Sweden comprising 196 questions is too long for widespread usage as a self-report questionnaire for screening oral health status in people with AN at medical centres and educational settings. It has been shown that people respond less readily to long questionnaires than short ones [31]. However, in our study, all the related oral health risk factors (behavioral and nutritional) and symptoms (salivary flow, gingival status, and dental status) were addressed in only 26 questions. The overall observed agreement between the SRQ-form compared with the face-to-face structured interview was relatively good. Irrational results regarding oral health perception and attitudes in people with eating disorders have been reported in the literature [14,16,32]. Indeed, denial is a frequent symptom in AN patients, especially among young people. Patients are known to give unreliable answers and/or deliberately omit talking about their somatic and psychological symptoms [32]. Some symptoms are perceived as shameful and therefore denied. In the present study, questions with the lowest agreements were those dealing with the purging/binge eating habits and the dental status. Consequently, the questions used to investigate these themes might be removed from a proposal of a short 10-item version of the questionnaire focused on the most reliable questions (Table 4). In addition, one of the most reliable self-reported items in our study, i.e., gingival bleeding, has been found to be a valid approach to determining gingival status in the general population and there is no reason to suspect that this would not hold true in the AN population [33].

Some limitations need to be acknowledged in our study. The young mean age of the patients limited the severity of the dental and periodontal manifestations, which increases with the duration of the eating disorder. However, this study focused on a sample of severely ill patients (hospitalized, with a very low BMI), among whom complications are frequent. There are many difficulties in populations with eating disorders in accessing a professional dental examination, mainly because of denial (patients) and the lack of awareness of oral health (physicians, psychiatrists). The questionnaire covers eating behaviors, physical activities, and dental symptoms that are not investigated routinely through dental/medical history. Despite the value of the questionnaire relying on its simplicity for self-administration by patients, the clinical implications and relevance for dental management need to be assessed. In addition, the questionnaire remains to be validated against clinical parameters for oral symptoms (salivary flow, gingival status, and dental status). Despite these limitations, this study developed the first reliable and easy to handle self-report questionnaire for evaluating risk factors and symptoms of oral disorders in anorexia nervosa. The results of this study suggest that a short version of the questionnaire would have value as a screening tool to benefit the AN patient’s awareness regarding the oral impact of their eating disorder and to initiate a conservation with the oral health professional for adequate prevention and treatment.

## 5. Conclusions

This study aimed to develop a specific questionnaire to assess oral risk factors and symptoms in anorexia nervosa. The hetero-administered form was designed as a guideline for dentists who are not trained in eating disorder care. On top of that, the short version (10-item) of the questionnaire has an additional value as a self-report form to prepare for a dental examination, and which should be part of the healthcare circuit of people with AN. Finally, this questionnaire could also be used for epidemiological studies in AN patients in order to identify the most frequent risk factors observed and associations with their mental and somatic clinical profile.

## Figures and Tables

**Table 1:** The 26-item questionnaire for screening oral risk factors and symptoms in anorexia nervosa.

Themes	Questions	Response’ Choices
Dental History (2 questions)	Q 1: How often did you have dental care?	Occasionally or Frequently
	Q2: What was the reason for dental treatment?	Scaling or Emergency or Check-up or Ongoing treatment
Lifestyle (8 questions)	Q3: Do you participate in sporting activities?	Yes or No
	Q4: For how long?	Less than 1 year or Between 1 to 4 years or More than 4 years
	Q5: How often?	Daily or On a regular basis or Occasionally
	Q6: What type of sporting activity do you practice?	Swimming-pool sport or Endurance sport or Dancing or Other sporting activity
	Q7: Are you used to monitoring your weight?	Daily or Occasionally or Never
	Q8: Have you noticed changes in your weight?	Yes or No
	Q9: In what direction?	Weight increase or Weight decrease?
	Q10: Are you satisfied with your body appearance?	Yes or No
Nutritional status (12 questions)	Q11: How many meals do you have per day?	1 or 2 or 3 or >3
	Q12: How often do you eat snack?	Daily or On a regular basis, Occasionally or Never
	Q13: Do you have the habit of fasting?	Frequently or periodically or Occasionally or Never
	Q14: Are you used to drinking sodas?	Yes or No
	Q15: Are you used to drinking fruit juice?	Yes or No
	Q16: Are you used to eating sweets?	Yes or No
	Q17: How much water do you drink per day?	Less than a bottle (1.5 L) or Close to a bottle (1.5 L) or More than a bottle (1.5 L)
Oral hygiene (4 questions)	Q18: How many times per day do you brush your teeth?	1 or 2 or 3 or >3
	Q19: What type of toothbrush do you use?	Soft bristles or Medium bristles or Hard bristles
Salivary flow (3 questions)	Q20: Do you often have a dry mouth?	Yes or No
	Q21: Do you have slimy saliva?	Yes or No
Gingival status (3 questions)	Q22: Do you have bleeding gums after toothbrushing?	Yes or No
	Q23: Have you noticed that your teeth are getting longer?	Yes or No
Dental status (4 questions)	Q24: Have you ever had any extracted teeth (except wisdom teeth)?	Yes or No
	Q25: Do you suffer from over-sensitive teeth?	Yes or No
	Q26: Do you think that the enamel of your teeth is damaged?	Yes or No

**Table 2:** Concordance analysis for each item between the self-report questionnaire and the face-to-face interview.

Items	Response’ Choices	With the Self-Report Questionnaire *n* (%)	During the Face-to-Face Interview *n* (%)	Kappa Coefficient	*p*-Value
Q 1: How often did you have dental care?	Occasionally	49 (87.5%)	49 (87.5%)	0.184	0.169
	Frequently	7 (12.5%)	7 (12.5%)		
Q2: What was the reason for dental treatment?	Scaling	11 (20.8%)	4 (7.5%)	0.184	0.042
	Emergency	1 (1.9%)	3 (5.7%)		
	Check-up	33 (62.3%)	36 (67.9%)		
	Ongoing treatment	8 (15.1%)	10 (18.9%)		
Q3: Do you participate in sporting activities?	No	22 (38.6%)	8 (14%)	0.244	0.023
	Yes	35 (61.4%)	49 (86%)		
Q4: For how long?	Less than 1 year	4 (13.8%)	3 (10.3%)	0.473	0.001
	Between 1 to 4 years	11 (37.9%)	10 (34.5%)		
	More than 4 years	14 (48.3%)	16 (55.2%)		
Q5: How often?	Daily	4 (13.8%)	4 (13.8%)	0.451	0.001
	On a regular basis	24 (82.8%)	21 (72.4%)		
	Occasionally	1 (3.4%)	4 (13.8%)		
Q6: What type of sporting activity do you practice?	Swimming-pool sport	4 (11.8%)	10 (29.4%)	0.474	<0.001
	Endurance sport	5 (14.7%)	4 (11.8%)		
	Dancing	10 (29.4%)	9 (26.5%)		
	Other sportive activity	15 (44.1%)	11 (32.4%)		
Q7: Are you used to monitoring your weight?	Daily	23 (41.8%)	30 (54.5%)	0.274	0.027
	Occasionally	31 (56.4%)	24 (43.6%)		
	Never	1 (1.8%)	1 (1.8%)		
Q8: Have you noticed changes in your weight?	No	12 (21.1%)	4 (7%)	0.022	0.841
	Yes	45 (78.9%)	53 (93%)		
Q9: In what direction?	Weight increase	33 (78.6%)	31 (73.8%)	0.215	0.16
	Weight decrease	9 (21.4%)	11 (26.2%)		
Q10: Are you satisfied with your body appearance?	No	29 (52.7%)	31 (56.4%)	0.487	<0.001
	Yes	26 (47.3%)	24 (43.6%)		
Q11: How many meals do you have per day?	2	3 (5.3%)	2 (3.5%)	0.36	0.002
	3	22 (38.6%)	21 (36.8%)		
	>3	32 (56.1%)	34 (59.6%)		
Q12: How often do you eat snack?	Daily	1 (1.8%)	2 (3.5%)	0.285	0.009
	On a regular basis	2 (3.5%)	2 (3.5%)		
	Occasionally	16 (28.1%)	13 (22.8%)		
	Never	38 (66.7%)	40 (70.2%)		
Q13: Do you have the habit of fasting?	Frequently	3 (55.5%)	2 (3.6%)	0.257	0.004
	Periodically	5 (9.1%)	4 (7.3%)		
	Occasionally	6 (10.9%)	14 (25.5%)		
	Never	41 (74.5%)	35 (63.6%)		
Q14: Are you used to drinking sodas?	No	52 (91.2%)	17 (29.8%)	0.026	0.615
	Yes	5 (8.8%)	40 (70.2%)		
Q15: Are you used to drinking fruit juice?	No	29 (50.9%)	7 (12.3%)	0.238	0.006
	Yes	28(49.1%)	50 (87.7%)		
Q16: Are you used to eating sweets?	No	47 (83.9%)	14 (25%)	0.12	0.059
	Yes	9 (16.1%)	42 (75%)		
Q17: How much water do you drink per day?	<1.5 L	22 (38.6%)	22 (38.6%)	0.655	<0.001
	around 1.5 L	30 (52.6%)	31 (54.4%)		
	>1.5 L	5 (8.8%)	4 (7%)		
Q18: How many times per day do you brush your teeth?	1	3 (5.3%)	2 (3.5%)	0.466	<0.001
	2	28 (49.1%)	33 (57.9%)		
	3	20 (35.1%)	19 (33.3%)		
	>3	6 (10.5%)	3 (5.3%)		
Q19: What type of toothbrush do you use?	Soft bristles	30 (54.5%)	20 (36.4%)	0.43	<0.001
	Medium bristles	23 (41.8%)	34 (61.8%)		
	Hard bristles	2 (3.6%)	1 (1.8%)		
Q20: Do you often have a dry mouth?	No	42 (73.7%)	38 (66.7%)	0.25	0.056
	Yes	15 (26.3%)	19 (33.3%)		
Q21: Do you have slimy saliva?	No	51 (91.1%)	53 (94.6%)	0.072	0.577
	Yes	5 (8.9%)	3 (5.4%)		
Q22: Do you have bleeding gums after toothbrushing?	No	39 (70.9%)	29 (52.7%)	0.479	<0.001
	Yes	16 (29.1%)	26 (47.3%)		
Q23: Have you noticed that your teeth are getting longer?	No	49 (86%)	53 (93%)	0.448	<0.001
	Yes	8 (14%)	4 (7%)		
Q24: Have you ever had any extracted teeth (except wisdom teeth)?	No	52 (92.9%)	51 (91.1%)	0.638	<0.001
	Yes	4 (7.1%)	5 (8.9%)		
Q25: Do you suffer from over-sensitive teeth?	No	36 (64.3%)	35 (62.5%)	0.346	0.01
	Yes	20 (35.7%)	21 (37.5%)		
Q26: Do you think that the enamel of your teeth is damaged?	No	48 (85.7%)	47 (83.9%)	0.376	0.005
	Yes	8 (14.3%)	9 (16.1%)		

**Table 3:** Reliability of the questionnaire’s items according to Landis and Koch [29].

Values of the Kappa Coefficient	Meaning of the Agreement	Items
≤0.2	slight	Q2
0.21–0.4	fair	Q3, Q7, Q11, Q12, Q13, Q15, Q25, Q26
0.41–0.6	moderate	Q4, Q5, Q6, Q10, Q18, Q19, Q22, Q23
>0.6	substantial	Q17, Q24

**Table 4:** Proposal for a short version of the self-report questionnaire based on the 10 most reliable items compared to the face-to-face interview.

Theme	Items	Questions
Lifestyle	Q short 1 (Q long-version 4)	For how long do you practice sporting activity?
	Q short 2 (Q long-version 5)	How often do you practice sporting activity?
	Q short 3 (Q long-version 6)	What type of sporting activity do you practice?
	Q short 4 (Q long-version 10)	Are you satisfied with your body appearance?
Nutritional status	Q short 5 (Q long-version 17)	How much water do you drink per day?
Oral hygiene	Q short 6 (Q long-version 18)	How many times per day do you brush your teeth?
	Q short 7 (Q long-version 19)	What type of toothbrush do you use?
Gingival status	Q short 8 (Q long-version 22)	Do you have bleeding gums after toothbrushing?
	Q short 9 (Q long-version 23)	Have you noticed that your teeth are getting longer?
Dental status	Q short 10 (Q long-version 24)	Have you ever had any extracted teeth (except wisdom teeth)?

## Data Availability

The data presented in this study are available on request from the corresponding author.
